# HDL Induces the Expression of the M2 Macrophage Markers Arginase 1 and Fizz-1 in a STAT6-Dependent Process

**DOI:** 10.1371/journal.pone.0074676

**Published:** 2013-08-21

**Authors:** Marie Sanson, Emilie Distel, Edward A. Fisher

**Affiliations:** The Leon H. Charney Division of Cardiology and Marc and Ruti Bell Program in Vascular Biology, Department of Medicine, New York University School of Medicine, New York, New York, United States of America; University of Virginia Health System, United States of America

## Abstract

Our lab has previously shown in a mouse model that normalization of a low HDL level achieves atherosclerotic plaque regression. This included the shift from a pro (“M1”) to an anti-inflammatory (“M2”) phenotypic state of plaque macrophages. Whether HDL can directly cause this phenotypic change and, if so, what the signaling mechanism is, were explored in the present studies. Murine primary macrophages treated with HDL showed increased gene expression for the M2 markers Arginase-1 (Arg-1) and Fizz-1, which are classically induced by IL-4. HDL was able to potentiate the IL-4-induced changes in Arg-1, and tended to do the same for Fizz-1, while suppressing the expression of inflammatory genes in response to IFNγ. The effects of either IL-4 or HDL were suppressed when macrophages were from STAT6^-/-^ mice, but inhibitor studies suggested differential utilization of JAK isoforms by IL-4 and HDL to activate STAT6 by phosphorylation. Overall, our results describe a new function of HDL, namely its ability to directly enrich macrophages in markers of the M2, anti-inflammatory, state in a process requiring STAT6.

## Introduction

Atherosclerosis is characterized by the infiltration of monocytes in the arterial wall that then become macrophages (for a recent review [1]). The clinical consequences of atherosclerosis include heart attacks and strokes, making it the leading cause of death in the western world. Our laboratory has introduced novel mouse models of atherosclerosis regression in order to learn strategies that could ultimately be applied to reversing the human disease [2,3]. With these models, we have observed a number of features of atherosclerotic plaques undergoing regression: 1) the decreased content of macrophages involves their emigration [4–6], which is consistent with the transcriptome analysis of the macrophages in regressing plaques showing significant changes in the expression of genes encoding cellular adhesion and cellular motility factors [7]; 2) Besides the change in the content of macrophages, their phenotype shifted from a pro- to an anti-inflammatory status based on M1 (inflammatory) and M2 (anti-inflammatory) macrophage markers [5,7]. Indeed, the highest up-regulated gene in macrophages in regressing plaques was found to be the M2 marker Arginase-1 (Arg-1); 3) the M2 shift was associated with increased plasma levels of high density lipoprotein (HDL), which was shown to be required [5].

Though HDL is known to have multiple protective mechanisms in atherosclerosis, the major effects have been thought to be its ability to remove excess cholesterol from cells, such as macrophages in arterial plaques that became foam cells. More recent studies have invoked anti-inflammatory properties (e.g., [8–11]), which may be related to the aforementioned finding that an increase in HDL resulted in a shift of plaque macrophages towards the M2 state. To extend that finding mechanistically, in the present report we have tested the hypothesis that HDL directly polarizes macrophages towards the M2 state, as would be reflected by the induction of the genes encoding Arg-1 and Fizz-1, two commonly accepted M2 markers in mice. If this turned out to be true, we were then interested in whether the effects depended on STAT6 signaling, which is required for the classical polarization of macrophages to the M2 state by IL-4 [12].

## Methods

### Mice

Wild-type C57Bl/6J and STAT6^-/-^ mice were purchased from The Jackson Laboratory. Experimental procedures were approved by the NYU School of Medicine Institutional Animal Care Committee.

### Reagents

HDL_3,_ the naturally occurring subfraction of spherical HDL typically used in cell culture studies, was isolated by sequential flotation by ultracentrifugation of plasma from healthy donor as previously described [13]. Hereafter, it is referred to simply as HDL. Endotoxin levels were assessed by the LAL assay (Enzo) and only endotoxin-free HDL preparations were used. Alternatively, endotoxin-free HDL was purchased from Kalen Biomedical LLC (Maryland). IL-4, IFNγ and M-CSF were purchased from Peprotech. The following antibodies were used: Phospho-STAT3 (Tyr705) and total-STAT3 (Cell Signaling), Phospho-STAT6 (Tyr641) (Imgenex), STAT6 (Santa Cruz Biotechnology Inc.), and GAPDH (Millipore). Kaempferol was purchased from Sigma Aldrich; Ruxolitinib and TG101348 were purchased from Selleck.

### Isolation and culture of bone marrow-derived macrophages (BMDM)

Bone marrow was isolated from the femur and tibia of 8-12 week old mice. After red blood cell lysis (RLB, Sigma Aldrich), cells were plated in Petri dishes in DMEM (1g/L glucose) containing 10% FBS and 12ng/mL M-CSF. 10ng/mL M-CSF was added on day 3, and on day 5, cells were seeded on tissue culture dishes with 8ng/mL M-CSF and treated on day 7. 16h before treatment, cells were serum-deprived in DMEM containing 0.2% free fatty acid BSA (Sigma-Aldrich). The treatments that the cells then underwent are described in the figure legends.

### Quantitative RT-qPCR

Total RNA was isolated from the BMDM using TRIzol according to the manufacturer (Invitrogen). 800ng of total RNA were reverse transcripted using the Verso cDNA kit (Thermo Scientific). Real-time PCR was performed using the ABI PRISM 7300 sequence detection system (Applied Biosystems). Gene expression was normalized to GAPDH expression and assessed using the Δ(ΔCt) calculation method. The following primers were designed with the Primer3 software: Arg1 (RE: TGGCTTGCGAGACGTAGAC; FW: GCTCAGGTGAATCGGCCTTTT), Fizz-1 (RE: GGTCCCAGTGCATATGGATGAGACC; FW: CACCTCTTCACTCGAGGGACAGTTG), GAPDH (RE: TGTAGACCATGTAGTTGAGGTCA; FW: AGGTCGGTGTGAACGGATTTG), IL-4R (RE: GCACCTGTGCATCCTGAATG; FW: TCTGCATCCCGTTGTTTTGC), IL-6 (RE: TTGGTCCTTAGCCACTCCTCC; FW: TAGTCCTTCCTACCCCAATTTCC), iNOS (RE: CTGATGGCAGACTACAAAGACG; FW: TGGCGGAGAGCATTTTTGAC), TNFα (RE: GCTACGACGTGGGCTACAG; FW: CCCTCACACTCAGATCATCTTCT).

### Western Blot

Proteins from BMDM were extracted in RIPA buffer containing anti-phosphatases PhosSTOP (Roche) and anti-proteases Complete (Roche). 10ug proteins were loaded on 8% polyacrylamide SDS gel. Transfer was done on nitrocellulose membrane. Primary antibodies were diluted at 1:2,500 and secondaries at 1:50,000. SuperSignal West Femto Chemiluminescent Substrate was used (Thermo scientific). Densitometry analysis of the films was carried out using Quantity One software (Biorad GS-800).

### Statistical analysis

Statistical analyses were performed using unpaired two-tailed t-test. This test was run using the Prism software (GraphPad). Results are displayed as mean ± SEM and were considered statistically significant when *p<0.05, **p<0.01, ***p<0.001 and ****p<0.0001.

## Results

### HDL promotes the alternative activation of primary murine macrophages in vitro

Real-time PCR performed on laser-captured macrophages from atherosclerotic plaques undergoing regression showed that several M2 markers, such as Arg-1, Mannose Receptor, CD163, Fizz-1 and YM-1 (e.g. [5,7]) were induced. Arg-1 is the enzyme that converts arginine to ornithine, a precursor of polyamines and proline. This conversion is critical in cell growth and wound healing [14]. The consumption of arginine by Arg-1 deprives inducible nitric oxide synthase (iNOS) from its substrate, and thus prevents the production of damagingly high levels of NO. This property of Arg-1 is thought to contribute to its immunosuppressive role in inflammatory loci [15,16]. Arg-1 is highly upregulated by IL-4 [17]. The function of Fizz-1 (Found in the inflammatory zone” and also referred as RELMα for Resistin-like molecule α) is less well defined. Fizz-1 was initially identified in models of murine asthmatic models [18] and has been implicated in mediating the deposition of extracellular matrix in an animal model of lung fibrosis and, thus, participating in wound healing and tissue remodeling like Arg-1 [19]. Also like Arg-1, Fizz-1 is specifically expressed by macrophages in response to IL-4 both *in vivo* and *in vitro* [20].

**Figure 1:**
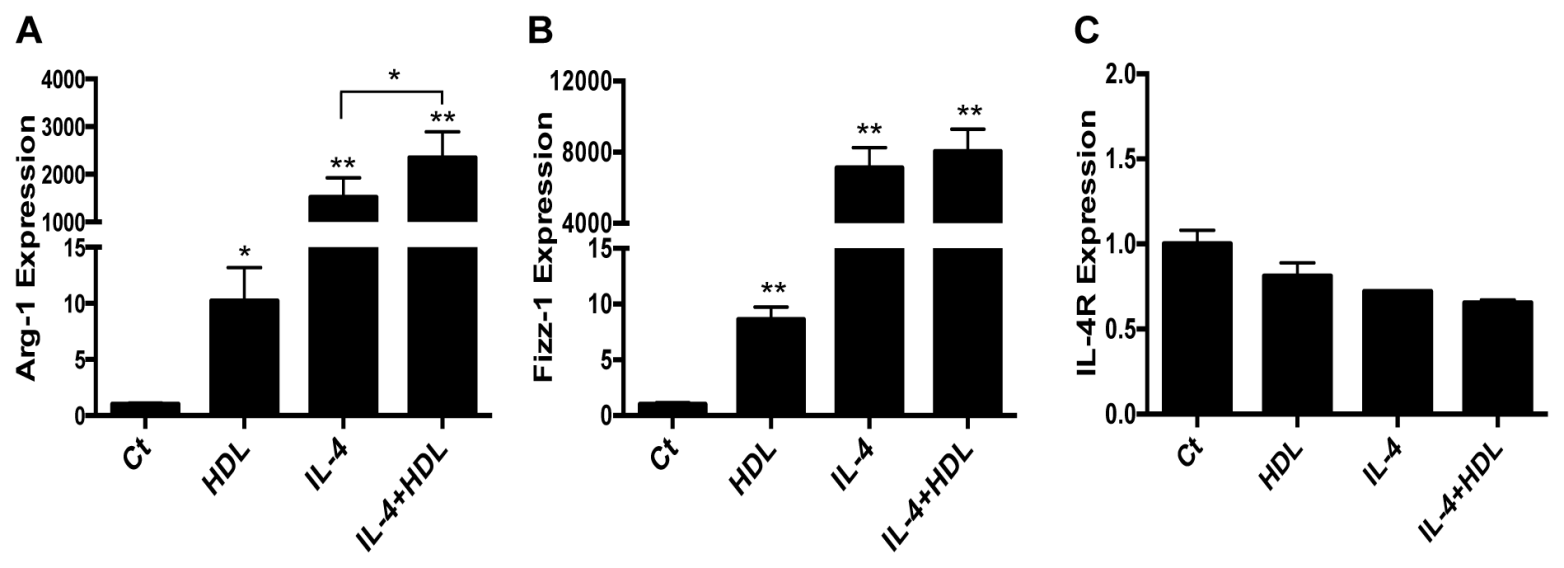
HDL promotes and enhances the expression of specific anti-inflammatory genes in primary macrophages. BMDM were treated for 6h with HDL (50μg/mL), IL-4 (10ng/mL) or both. Gene expression of Arg-1 (A), Fizz-1 (B) and IL-4R (C) was assessed by real-time qPCR. Results are representative of four independent experiments. Asterisks indicate statistically significant differences, either compared to control, or between 2 conditions when linked by a bar (*p<0.05, **p<0.01).

In the first set of experiments, BMDM in the resting (“M0”) state were treated with HDL at a concentration attained in tissues. As shown in Figure 1, this resulted in upregulation by ~10 fold of Arg-1 and Fizz-1 gene expression, as we previously published [5]. Notably, co-treatment with IL-4 and HDL resulted in the highest level of either Arg-1 or Fizz-1 gene expression, with 826 or 919-fold increases, respectively, over the level observed with IL-4 alone, though this additive effect was statistically significant only for Arg-1 (Figure 1A, B). This statistical difference could represent either variations in the regulation of these two M2 markers, or it could be due to the fact that Fizz-1 induction is already much higher with IL-4 than that of Arg-1. The effects of HDL or IL-4 appeared to be specific, both as single agents and in combination, based on the lack of induction of IL-4 receptor α (Figure 1C), which is required for M2 polarization by IL-4, but is not a STAT6-dependent target.

**Figure 2:**
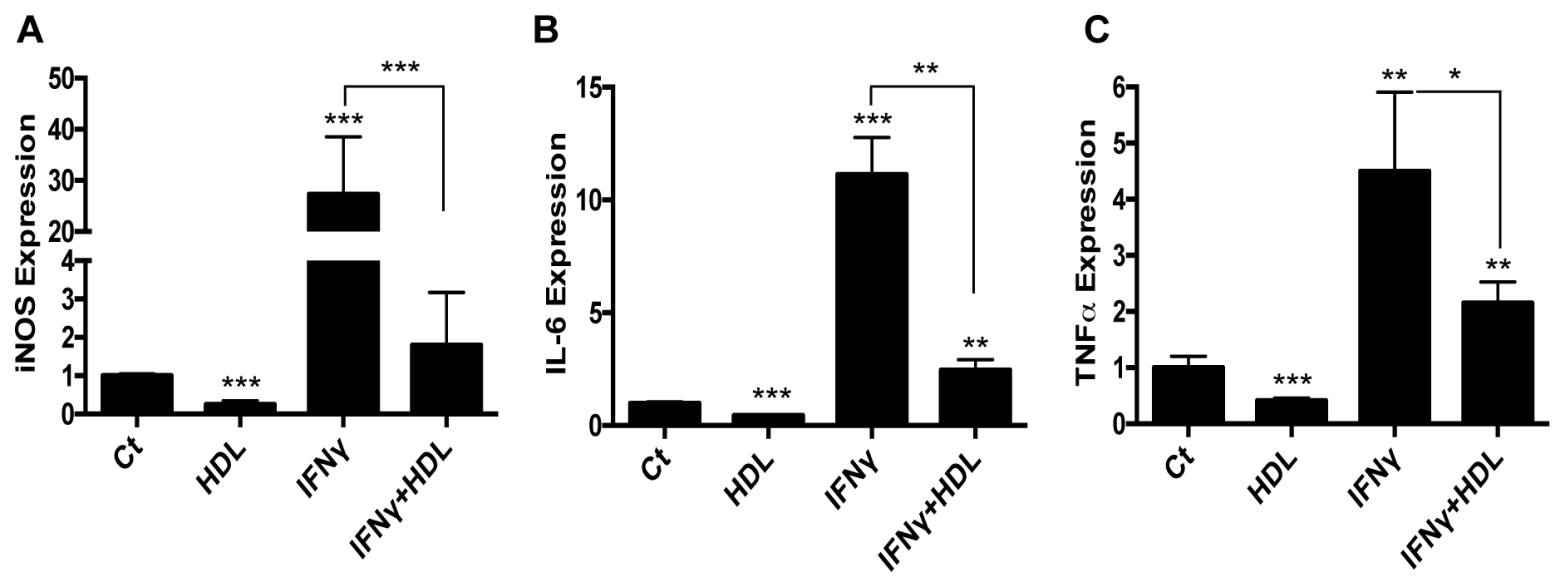
HDL inhibits the basal and induced expression of pro-inflammatory genes in primary macrophages. BMDM were treated for 6h with HDL (50μg/mL), IFNγ (10ng/mL) or both. Gene expression for iNOS (A), IL-6 (B) or TNFα (C) was assessed by real-time qPCR. Results are representative of three independent experiments. Asterisks indicate statistically significant differences, either compared to control, or between 2 conditions when linked by a bar (*p<0.05, **p<0.01, ***p<0.001).

HDL is known to prevent pro-inflammatory activation of monocytes and macrophages (e.g., [8–11,21]). Thus, we hypothesized that if HDL were able to promote enrichment in M2 markers, it may also inhibit the gene expression of M1 markers. Indeed, Figure 2 shows that HDL is able to suppress basal and IFNγ-induced expression of M1 markers, such as iNOS (Figure 2A), IL-6 (Figure 2B) and TNF-α (Figure 2C). To our knowledge, these results show for the first time that HDL can directly induce the expression of M2 markers, as well as potentiate effects of IL-4, while also inhibiting M1 markers.

### HDL promotes phosphorylation of STAT6, but not STAT3

**Figure 3:**
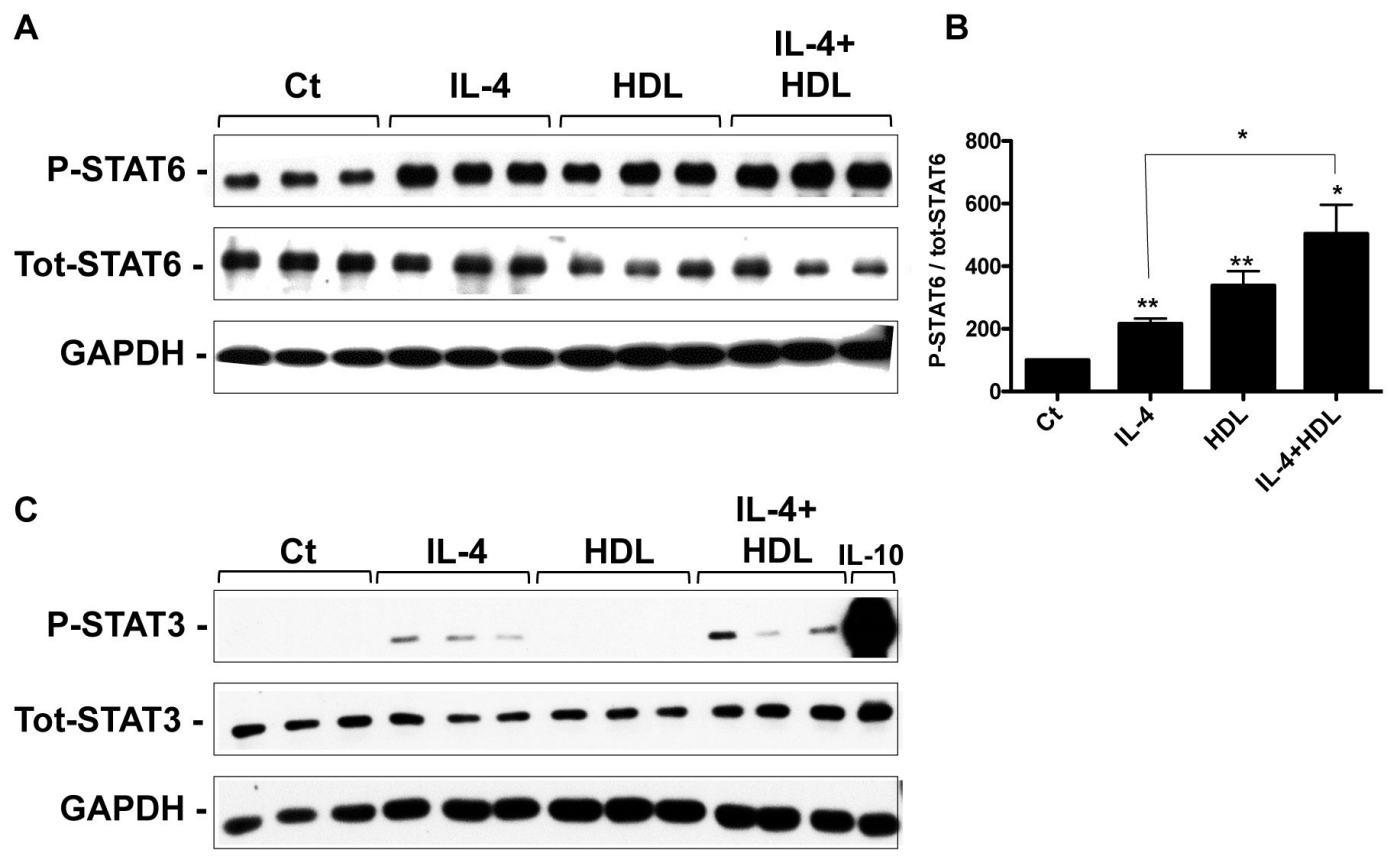
HDL induces STAT6 phosphorylation and enhances IL-4-induced STAT6 activation, but has no effect on STAT3 phosphorylation status. BMDM were grown as previously described and stimulated for 15min with either HDL alone (50μg/mL), IL-4 alone (10ng/mL) or both. Phosphorylation state for STAT6 (A) and STAT3 (C) was assessed by western blot. As a positive control for STAT3 activation, cells were treated with IL-10 (10ng/mL) for 30 min. Signal quantification for phospho-STAT6 (B) is the result of three independent experiments. Asterisks indicate statistically significant difference, either compared to control, or between 2 conditions when linked by a bar (*p<0.05, **p<0.01).

The classical pathway for IL-4-induced polarization involves the activation of the STAT6 (Signal Transducer and Activator of Transcription) signaling pathway [22] and STAT6 is one of the trans factors that bind to the promoter of Arg-1 and Fizz-1 [17,23]. As HDL activates Arg-1 and Fizz-1 gene expression (Figure 1), we investigated whether these effects were mediated by the STAT6 signaling pathway. As a general mechanism, STAT proteins are activated by phosphorylation by the Janus Kinases (JAK1/2/3) family. This process is rapid upon cytokine receptor binding. As shown in Figure 3A and 3B, within 15 min, both IL-4 and HDL similarly induced the phosphorylation of STAT6 compared to the non-treated condition. While IL-4 was able to sustain the phosphorylation of STAT6 for up to 4h, the HDL effect was shorter and started to decrease after 30min (data not shown), which could account for the less potent effects of HDL on Arg-1 and Fizz-1 (Figure 1A and B). The combination of HDL and IL-4, however, further enhanced the phosphorylation of STAT6 compared to IL-4 treatment alone (p<0.05; Figure 3B), which may explain why the highest levels of Arg-1 and Fizz-1 gene expression were observed when both agents were used in combination (Figure 1A and B). It is interesting to note that the changes in STAT6 phosphorylation (Figure 3) were not quantitatively equivalent to the corresponding changes in gene expression (Figure 1). This is consistent with other studies (e.g., [24]), and may represent either amplification of effects downstream of STAT6, the selection of non-optimal time points for analysis, or the semi-quantitative nature of western blotting.

Turning to STAT3, other groups have shown its involvement in the signaling pathway induced by HDL and ApoA1, HDL’s most abundant protein. For example, STAT3 is activated in macrophages upon ApoA1 binding to the cholesterol transporter ABCA1 [9]. Also, in cardiomyocytes, HDL induces the phosphorylation of STAT3 on both tyrosine 705 and serine 727 [25]. To investigate whether the effects of HDL on Arg-1 and Fizz-1 expression in macrophages involved STAT3 activation, the same type of experiment conducted to assess STAT6 phosphorylation was performed. The canonical pathway for STAT3 activation is induced by IL-10 [26], so cells were treated with this cytokine as a positive control. As previously shown in other cell types, IL-4 was able to induce the phosphorylation of STAT3 [27]. However, and in contrast to what has been published [25], HDL failed to activate STAT3 either alone or in combination with IL-4 (Figure 3C), suggesting that this effect may be tissue-specific. This last result is also consistent with the lack of effect of HDL on IL-4r expression (Figure 1C), as IL-4r expression is regulated by the IL-10/STAT3 pathway [28].

Overall, the results suggest that in BMDM, STAT6, but not STAT3, is involved in the induction of Arg-1 and Fizz-1 by HDL. In addition, to our knowledge, the results show for the first time the ability of HDL to promote STAT6 phosphorylation.

### STAT6 deficiency impairs the induction of Arg-1 and Fizz-1 by either HDL or IL-4

**Figure 4:**
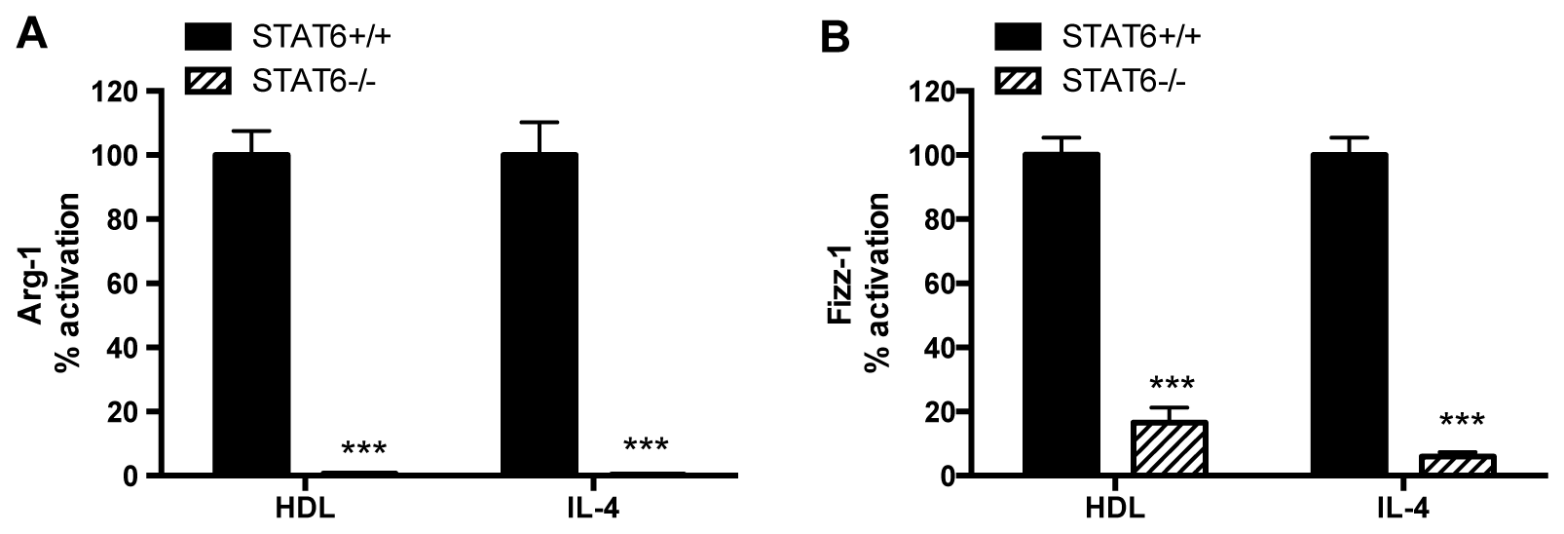
STAT6 inhibition affects macrophages alternative activation by HDL. BMDM were isolated from STAT6^+/+^ (black bars) and STAT6-/- (hatched bars) mice and were stimulated for 6h with HDL (50μg/mL) or IL-4 (10ng/mL). The induction of Arg-1 or Fizz-1 mRNA in the STAT6+/+ cells by HDL was ~3X for each and by IL-4, ~1800 or 3300, respectively; these values were set to 100%. Results are representative of three independent experiments. Asterisks indicate statistically significant differences compared to corresponding STAT6^+/+^ conditions (***p<0.001).

The fact that STAT6 phosphorylation is promoted by HDL suggests, but does not prove, that this is required for HDL’s effects on Arg-1 and Fizz-1. To directly test this, we examined Arg-1 and Fizz-1 gene expression under conditions where the STAT6 pathway was inhibited genetically by isolating BMDM from STAT6^-/-^ mice. As shown in Figure 4A and B and, as expected, Arg-1 and Fizz-1 induction by IL-4 was significantly blunted by deficiency of STAT6, but notably, so was the induction by HDL. As noted above, STAT6 phosphorylation is mediated by JAK kinases, of which there are 3 isoforms: 1, 2 and 3. Ruxolitinib and TG101348 are chemical inhibitors of Jak1/2 and Jak2, respectively. Kaempferol is a natural polyphenol compound that has been shown to specifically block IL-4-induced STAT6 phosphorylation through the selective inhibition of JAK3 activity [29]. The canonical pathway for IL-4 induced STAT6 phosphorylation typically involves JAK1 and JAK3 [30], though JAK2 can also participate (e.g., [31]).

**Figure 5:**
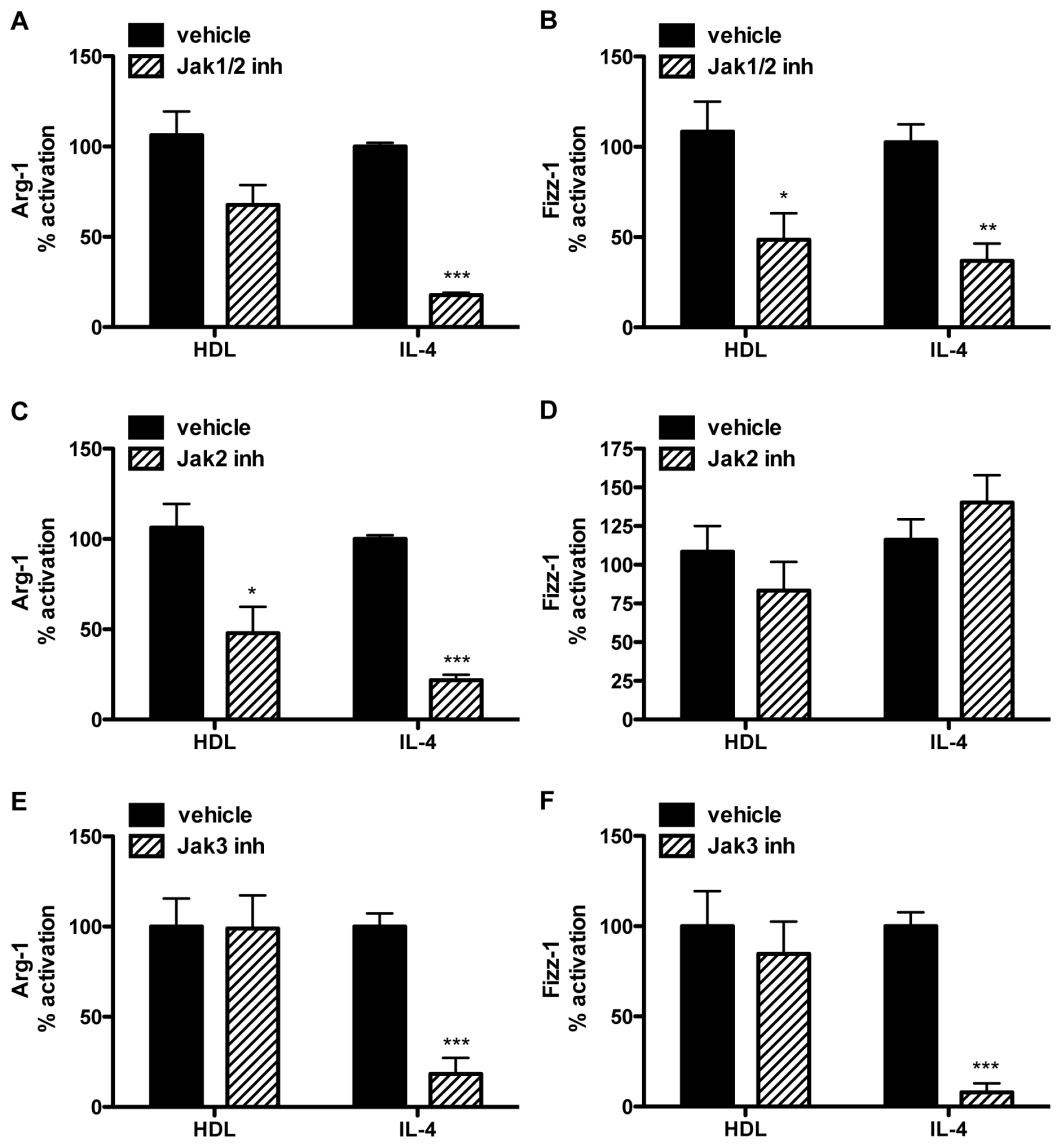
Jak inhibition affects macrophages alternative activation by HDL. BMDM were pre-treated for 2h with Jak pharmacological inhibitors (hatched bars. Ruxolitinib/Jak1/2 inhibitor: 1μM. TG101348/Jak2 inhibitor: 1μM: Kaempferol/Jak3 inhibitor: 40μM) or vehicle (black bars), and then treated for 6h with HDL (50μg/mL) or IL-4 (10ng/mL). Arg-1 (A, C, E) and Fizz-1 (B, D, F) expression are presented as the percentage of activation compared to non-inhibited conditions. The induction of Arg-1 and Fizz-1 by HDL was ~2X for each, and for IL-4 was >300 for Arg-1 and Fizz-1 and these values were set to 100%. Results are representative of three independent experiments. Asterisks indicate statistically significant differences compared to the vehicle-treated conditions (*p<0.05, **p<0.01, ***p<0.001).

For IL-4’s induction of Arg-1 and Fizz-1, the data show that either the JAK1/2 (Ruxolinib) or JAK3 (Kaempferol) inhibitor blunted the effect (Figure 5). The JAK2 inhibitor (TG101348) impaired IL-4-induced Arg-1, but not Fizz-1 expression, implying that the effects of the JAK1/2 inhibitor on Arg-1 and Fizz-1 induction were attributable to its anti-JAK2 and anti-JAK1 activities, respectively. For HDL, its induction of Arg-1 appeared to involve mainly JAK2. In contrast, the most potent effect on HDL induction of Fizz-1was attributable to JAK1, given the significant decrease with the JAK1/2, but not the JAK2 or JAK3, inhibitor.

Overall, the results in Figures 4 and 5 suggest that the effects of HDL, and, as expected, of IL-4 on Arg-1 and Fizz-1 gene expression require STAT6, but that the pattern of kinases used for its activation differs not only by inducing agent, but also by gene, again highlighting variations in the regulation of Arg-1 and Fizz-1.

## Discussion

Macrophages are central to atherosclerotic progression and regression, and their inflammatory state greatly influences the course of the disease [32]. HDL is thought to dampen macrophage responses to pro-inflammatory stimuli [8–10]. This property of HDL is often attributed to its capacity to promote cholesterol efflux, which could re-organize micro-domains of the plasma membrane, thereby affecting the functional properties of components of inflammatory pathways (e.g., the TLRs) [8,10]. Because a number of proteins [33] and lipids [34] carried on HDL particles may also influence inflammation, there may be other mechanisms by which HDL blunts the responses of activated macrophages (termed M1) in the plaque.

In addition to blunting macrophage activation, which we have also observed (Figure 2), another potential anti-inflammatory property of HDL to consider is the promotion of macrophage polarization to the “alternatively activated” M2 state. M2 macrophages not only are low producers of inflammatory substances, such as IL-6 and TNFα, they are high producers of IL-10, a most potent anti-inflammatory cytokine [35]. The present study shows for the first time that HDL is able to drive macrophages *in vitro* towards an anti-inflammatory state, as shown by the induction of two major M2 markers, Arg-1 and Fizz-1. These effects depended on STAT6, which is also employed by a classical inducer of the M2 state, IL-4 [22]. Thus, HDL simultaneously inhibits the basal and IFNγ-induced expression (often STAT1-dependent [36]) of M1 markers, such as iNOS, IL-6 and TNFα. Antagonism between the STAT1 and STAT3 pathways is well described, notably in cardiovascular diseases [37], but reciprocal regulation between STAT1 and STAT6 is an emerging field [38], and our results may be reflecting such a phenomenon mediated by HDL with regard to macrophage inflammatory state.

The present results also showed that HDL can potentiate the effect of IL-4 on Arg-1. This potentiation by HDL can be most simply explained by the increase in IL-4-induced STAT6 phosphorylation (which reflects its activation) when both factors were used to treat cells, despite Fizz-1 induction not being similarly affected (though it tended to be highest in the combined IL-4/HDL condition). The fact that the combined effects of HDL and IL-4 on STAT6 phosphorylation were greater than either alone suggested that, each one could be stimulating STAT6 phosphorylation by different mechanisms. This was supported by studies using selective JAK inhibitors indicating that, while IL-4 mainly activates JAK1 and JAK3, HDL likely induces the activation of JAK1 and JAK2.

We also investigated whether the effects of HDL were through STAT3, because work by Oram and colleagues had shown for the major protein component of HDL, apoAI, that when it is in a lipid-poor state it can stimulate STAT3 phosphorylation through interaction with ABCA1 [9,39]. STAT3 has also been linked indirectly to M2 polarization by being able to induce expression of a subunit of the IL-4 receptor [28]. As shown in Figure 3, however, we found no evidence that HDL promotes STAT3 phosphorylation, despite its containing apoAI. The simplest explanation for this is that typical HDL particles (e.g., HDL3) do not interact significantly with ABCA1 because the conformation of apoAI on such a spherical particle is far different from what it is in the lipid-poor state [40].

It is interesting to imagine how HDL modulates the STAT6 phosphorylation required for the induction of the genes encoding the M2 markers Arg-1 and Fizz-1. Based on work from the laboratories of Alan Tall and John Parks on HDL effects on TLR signaling [10,11], it is likely related to the interaction of HDL with the plasma membrane removing cholesterol through transporter dependent and independent mechanisms. This results in changes in membrane micro-domains, particularly in lipid rafts that serve as signaling platforms where many proteins are anchored. Indeed, the activation state and responsiveness of some of these proteins have been shown to be significantly affected by lipid raft modification due to cholesterol efflux from the plasma membrane [10,11]. Particularly relevant to the present findings is the ‘raft-STAT signaling’ hypothesis, in which Patel and colleagues have provided data that an appreciable percent of total cytoplasmic STATs are associated with lipid rafts, suggesting that this pool is more likely to display altered activation whenever its membrane micro-environment is affected [41].

Consistent with these considerations are preliminary data from experiments using BMDM prepared from ABCG1-deficient mice. ABCG1 preferentially facilitates cholesterol efflux to HDL particles (vs. the ABCA1/ApoAI-mediated efflux pathway), and the induction of Arg-1 expression by HDL was inhibited by ~75% in ABCG1^-/-^ BMDM. That efflux-related phenomena were relevant to the present results is also supported by the natural cholesterol-loading of bone marrow-derived cells that occurs upon incubation with 10% FBS during their differentiation *in vitro* [42]. When these cells are then exposed to HDL, cholesterol efflux would rapidly commence and continue during the treatment period.

In summary, *in vivo* and *in vitro* studies have emphasized the protective role of HDL in atherosclerosis, but recent clinical studies have emphasized it is not the level of HDL cholesterol that is necessarily beneficial, it is the functionality of the HDL particles [43,44]. Our results extend HDL functionality into a novel direction and provide a framework to delve deeper into the associated mechanisms. In addition, the work by us [5,45] and others [46] that M2 macrophages are associated both with atherosclerosis regression and delayed progression in mouse models, suggest that the role of HDL in macrophage polarization is likely to be clinically relevant.

## References

[1] Moore KJ, Tabas I (2011) Macrophages in the pathogenesis of atherosclerosis. Cell 145: 341-355. doi:10.1016/j.cell.2011.04.005. PubMed: 21529710. 21529710 10.1016/j.cell.2011.04.005 PMC3111065

[2] Feig JE, Quick JS, Fisher EA (2009) The role of a murine transplantation model of atherosclerosis regression in drug discovery. Curr Opin Investig Drugs 10: 232-238. PubMed: 19333880. PMC4662935 19333880

[3] Reis ED, Li J, Fayad ZA, Rong JX, Hansoty D et al. (2001) Dramatic remodeling of advanced atherosclerotic plaques of the apolipoprotein E-deficient mouse in a novel transplantation model. J Vasc Surg 34: 541-547. doi:10.1067/mva.2001.115963. PubMed: 11533609. 11533609 10.1067/mva.2001.115963

[4] Llodrá J, Angeli V, Liu J, Trogan E, Fisher EA et al. (2004) Emigration of monocyte-derived cells from atherosclerotic lesions characterizes regressive, but not progressive, plaques. Proc Natl Acad Sci U S A 101: 11779-11784. doi:10.1073/pnas.0403259101. PubMed: 15280540. 15280540 10.1073/pnas.0403259101 PMC511052

[5] Feig JE, Rong JX, Shamir R, Sanson M, Vengrenyuk Y et al. (2011) HDL promotes rapid atherosclerosis regression in mice and alters inflammatory properties of plaque monocyte-derived cells. Proc Natl Acad Sci U S A 108: 7166-7171. doi:10.1073/pnas.1016086108. PubMed: 21482781. 21482781 10.1073/pnas.1016086108 PMC3084076

[6] Trogan E, Feig JE, Dogan S, Rothblat GH, Angeli V et al. (2006) Gene expression changes in foam cells and the role of chemokine receptor CCR7 during atherosclerosis regression in ApoE-deficient mice. Proc Natl Acad Sci U S A 103: 3781-3786. doi:10.1073/pnas.0511043103. PubMed: 16537455. 16537455 10.1073/pnas.0511043103 PMC1450154

[7] Feig JE, Vengrenyuk Y, Reiser V, Wu C, Statnikov A et al. (2012) Regression of atherosclerosis is characterized by broad changes in the plaque macrophage transcriptome. PLOS ONE 7: e39790. doi:10.1371/journal.pone.0039790. PubMed: 22761902. 22761902 10.1371/journal.pone.0039790 PMC3384622

[8] Murphy AJ, Woollard KJ, Hoang A, Mukhamedova N, Stirzaker RA et al. (2008) High-density lipoprotein reduces the human monocyte inflammatory response. Arterioscler Thromb Vasc Biol 28: 2071-2077. doi:10.1161/ATVBAHA.108.168690. PubMed: 18617650. 18617650 10.1161/ATVBAHA.108.168690

[9] Tang C, Liu Y, Kessler PS, Vaughan AM, Oram JF (2009) The macrophage cholesterol exporter ABCA1 functions as an anti-inflammatory receptor. J Biol Chem 284: 32336-32343. doi:10.1074/jbc.M109.047472. PubMed: 19783654. 19783654 10.1074/jbc.M109.047472 PMC2781648

[10] Yvan-Charvet L, Welch C, Pagler TA, Ranalletta M, Lamkanfi M et al. (2008) Increased inflammatory gene expression in ABC transporter-deficient macrophages: free cholesterol accumulation, increased signaling via toll-like receptors, and neutrophil infiltration of atherosclerotic lesions. Circulation 118: 1837-1847. doi:10.1161/CIRCULATIONAHA.108.793869. PubMed: 18852364. 18852364 10.1161/CIRCULATIONAHA.108.793869 PMC2756536

[11] Zhu X, Lee JY, Timmins JM, Brown JM, Boudyguina E et al. (2008) Increased cellular free cholesterol in macrophage-specific Abca1 knock-out mice enhances pro-inflammatory response of macrophages. J Biol Chem 283: 22930-22941. doi:10.1074/jbc.M801408200. PubMed: 18552351. 18552351 10.1074/jbc.M801408200 PMC2516976

[12] Mandal P, Pratt BT, Barnes M, McMullen MR, Nagy LE (2011) Molecular mechanism for adiponectin-dependent M2 macrophage polarization: link between the metabolic and innate immune activity of full-length adiponectin. J Biol Chem 286: 13460-13469. doi:10.1074/jbc.M110.204644. PubMed: 21357416. 21357416 10.1074/jbc.M110.204644 PMC3075692

[13] Hatch FT (1968) Practical methods for plasma lipoprotein analysis. Adv Lipid Res 6: 1-68. PubMed: 4179999. 4179999

[14] Shearer JD, Richards JR, Mills CD, Caldwell MD (1997) Differential regulation of macrophage arginine metabolism: a proposed role in wound healing. Am J Physiol 272: E181-E190. PubMed: 9124321. 9124321 10.1152/ajpendo.1997.272.2.E181

[15] Khallou-Laschet J, Varthaman A, Fornasa G, Compain C, Gaston AT et al. (2010) Macrophage plasticity in experimental atherosclerosis. PLOS ONE 5: e8852. doi:10.1371/journal.pone.0008852. PubMed: 20111605. 20111605 10.1371/journal.pone.0008852 PMC2810335

[16] Thomas AC, Sala-Newby GB, Ismail Y, Johnson JL, Pasterkamp G et al. (2007) Genomics of foam cells and nonfoamy macrophages from rabbits identifies arginase-I as a differential regulator of nitric oxide production. Arterioscler Thromb Vasc Biol 27: 571-577. doi:10.1161/01.ATV.0000256470.23842.94. PubMed: 17194896. 17194896 10.1161/01.ATV.0000256470.23842.94

[17] Gray MJ, Poljakovic M, Kepka-Lenhart D, Morris SM Jr. (2005) Induction of arginase I transcription by IL-4 requires a composite DNA response element for STAT6 and C/EBPbeta. Gene 353: 98-106. doi:10.1016/j.gene.2005.04.004. PubMed: 15922518. 15922518 10.1016/j.gene.2005.04.004

[18] Holcomb IN, Kabakoff RC, Chan B, Baker TW, Gurney A et al. (2000) FIZZ1, a novel cysteine-rich secreted protein associated with pulmonary inflammation, defines a new gene family. EMBO J 19: 4046-4055. doi:10.1093/emboj/19.15.4046. PubMed: 10921885. 10921885 10.1093/emboj/19.15.4046 PMC306596

[19] Liu T, Dhanasekaran SM, Jin H, Hu B, Tomlins SA et al. (2004) FIZZ1 stimulation of myofibroblast differentiation. Am J Pathol 164: 1315-1326. doi:10.1016/S0002-9440(10)63218-X. PubMed: 15039219. 15039219 10.1016/S0002-9440(10)63218-X PMC1615359

[20] Raes G, Noël W, Beschin A, Brys L, de Baetselier P et al. (2002) FIZZ1 and Ym as tools to discriminate between differentially activated macrophages. Dev Immunol 9: 151-159. doi:10.1080/1044667031000137629. PubMed: 12892049. 12892049 10.1080/1044667031000137629 PMC2276098

[21] Murphy AJ, Akhtari M, Tolani S, Pagler T, Bijl N et al. (2011) ApoE regulates hematopoietic stem cell proliferation, monocytosis, and monocyte accumulation in atherosclerotic lesions in mice. J Clin Invest 121: 4138-4149. doi:10.1172/JCI57559. PubMed: 21968112. 21968112 10.1172/JCI57559 PMC3195472

[22] Nelms K, Keegan AD, Zamorano J, Ryan JJ, Paul WE (1999) The IL-4 receptor: signaling mechanisms and biologic functions. Annu Rev Immunol 17: 701-738. doi:10.1146/annurev.immunol.17.1.701. PubMed: 10358772. 10358772 10.1146/annurev.immunol.17.1.701

[23] Stütz AM, Pickart LA, Trifilieff A, Baumruker T, Prieschl-Strassmayr E et al. (2003) The Th2 cell cytokines IL-4 and IL-13 regulate found in inflammatory zone 1/resistin-like molecule alpha gene expression by a STAT6 and CCAAT/enhancer-binding protein-dependent mechanism. J Immunol 170: 1789-1796. PubMed: 12574343. 12574343 10.4049/jimmunol.170.4.1789

[24] Lu X, Malumbres R, Shields B, Jiang X, Sarosiek KA et al. (2008) PTP1B is a negative regulator of interleukin 4-induced STAT6 signaling. Blood 112: 4098-4108. doi:10.1182/blood-2008-03-148726. PubMed: 18716132. 18716132 10.1182/blood-2008-03-148726 PMC2582009

[25] Frias MA, James RW, Gerber-Wicht C, Lang U (2009) Native and reconstituted HDL activate Stat3 in ventricular cardiomyocytes via ERK1/2: role of sphingosine-1-phosphate. Cardiovasc Res 82: 313-323. 19151362 10.1093/cvr/cvp024

[26] Murray PJ (2006) Understanding and exploiting the endogenous interleukin-10/STAT3-mediated anti-inflammatory response. Curr Opin Pharmacol 6: 379-386. doi:10.1016/j.coph.2006.01.010. PubMed: 16713356. 16713356 10.1016/j.coph.2006.01.010

[27] Rolling C, Treton D, Pellegrini S, Galanaud P, Richard Y (1996) IL4 and IL13 receptors share the gamma c chain and activate STAT6, STAT3 and STAT5 proteins in normal human B cells. FEBS Lett 393: 53-56. 8804422 10.1016/0014-5793(96)00835-6

[28] Biswas A, Bhattacharya A, Kar S, Das PK (2011) Expression of IL-10-triggered STAT3-dependent IL-4Ralpha is required for induction of arginase 1 in visceral leishmaniasis. Eur J Immunol 41: 992-1003. doi:10.1002/eji.201040940. PubMed: 21413004. 21413004 10.1002/eji.201040940

[29] Cortes JR, Perez GM, Rivas MD, Zamorano J (2007) Kaempferol inhibits IL-4-induced STAT6 activation by specifically targeting JAK3. J Immunol 179: 3881-3887. 17785825 10.4049/jimmunol.179.6.3881

[30] Wurster AL, Tanaka T, Grusby MJ (2000) The biology of Stat4 and Stat6. Oncogene 19: 2577-2584. doi:10.1038/sj.onc.1203485. PubMed: 10851056. 10851056 10.1038/sj.onc.1203485

[31] Choi H, Choi H, Han J, Jin SH, Park JY et al. (2013) IL-4 inhibits the melanogenesis of normal human melanocytes through the JAK2-STAT6 signaling pathway. J Invest Dermatol 133: 528-536. doi:10.1038/jid.2012.331. PubMed: 22992805. 22992805 10.1038/jid.2012.331

[32] Williams HJ, Fisher EA, Greaves DR (2012) Macrophage differentiation and function in atherosclerosis: opportunities for therapeutic intervention? J Innate Immun 4: 498-508. doi:10.1159/000336618. PubMed: 22572544. 22572544 10.1159/000336618 PMC3598573

[33] Vaisar T, Pennathur S, Green PS, Gharib SA, Hoofnagle AN et al. (2007) Shotgun proteomics implicates protease inhibition and complement activation in the antiinflammatory properties of HDL. J Clin Invest 117: 746-756. doi:10.1172/JCI26206. PubMed: 17332893. 17332893 10.1172/JCI26206 PMC1804352

[34] Egom EE, Mamas MA, Soran H (2013) HDL quality or cholesterol cargo: what really matters - spotlight on sphingosine-1-phosphate-rich HDL. Curr Opin Lipidol, 24: 351–6. PubMed: 23652570. 23652570 10.1097/MOL.0b013e328361f822

[35] Sica A, Mantovani A (2012) Macrophage plasticity and polarization: in vivo veritas. J Clin Invest 122: 787-795. doi:10.1172/JCI59643. PubMed: 22378047. 22378047 10.1172/JCI59643 PMC3287223

[36] Ramana CV, Gil MP, Schreiber RD, Stark GR (2002) Stat1-dependent and -independent pathways in IFN-gamma-dependent signaling. Trends Immunol 23: 96-101. doi:10.1016/S1471-4906(01)02118-4. PubMed: 11929133. 11929133 10.1016/s1471-4906(01)02118-4

[37] Albasanz-Puig A, Murray J, Namekata M, Wijelath ES (2012) Opposing roles of STAT-1 and STAT-3 in regulating vascular endothelial growth factor expression in vascular smooth muscle cells. Biochem Biophys Res Commun 428: 179-184. doi:10.1016/j.bbrc.2012.10.037. PubMed: 23068100. 23068100 10.1016/j.bbrc.2012.10.037

[38] Miyamoto H, Katsuyama E, Miyauchi Y, Hoshi H, Miyamoto K et al. (2012) An essential role for STAT6-STAT1 protein signaling in promoting macrophage cell-cell fusion. J Biol Chem 287: 32479-32484. doi:10.1074/jbc.M112.358226. PubMed: 22865856. 22865856 10.1074/jbc.M112.358226 PMC3463345

[39] Liu Y, Tang C (2012) Regulation of ABCA1 functions by signaling pathways. Biochim Biophys Acta 1821: 522-529. doi:10.1016/j.bbalip.2011.08.015. PubMed: 21920460. 21920460 10.1016/j.bbalip.2011.08.015 PMC3243790

[40] Vaughan AM, Oram JF (2005) ABCG1 redistributes cell cholesterol to domains removable by high density lipoprotein but not by lipid-depleted apolipoproteins. J Biol Chem 280: 30150-30157. doi:10.1074/jbc.M505368200. PubMed: 15994327. 15994327 10.1074/jbc.M505368200

[41] Sehgal PB, Guo GG, Shah M, Kumar V, Patel K (2002) Cytokine signaling: STATS in plasma membrane rafts. J Biol Chem 277: 12067-12074. doi:10.1074/jbc.M200018200. PubMed: 11815625. 11815625 10.1074/jbc.M200018200

[42] Sankaranarayanan S, de la Llera-Moya M, Drazul-Schrader D, Asztalos BF, Weibel GL et al. (2010) Importance of macrophage cholesterol content on the flux of cholesterol mass. J Lipid Res 51: 3243-3249. doi:10.1194/jlr.M008441. PubMed: 20713652. 20713652 10.1194/jlr.M008441 PMC2952564

[43] Rader DJ, Tall AR (2012) The not-so-simple HDL story: Is it time to revise the HDL cholesterol hypothesis? Nat Med 18: 1344-1346. doi:10.1038/nm.2937. PubMed: 22961164. 22961164 10.1038/nm.2937

[44] Hewing B, Moore KJ, Fisher EA (2012) HDL and cardiovascular risk: time to call the plumber? Circ Res 111: 1117-1120. doi:10.1161/CIRCRESAHA.112.280958. PubMed: 23065341. 23065341 10.1161/CIRCRESAHA.112.280958 PMC3617479

[45] Feig JE, Parathath S, Rong JX, Mick SL, Vengrenyuk Y et al. (2011) Reversal of hyperlipidemia with a genetic switch favorably affects the content and inflammatory state of macrophages in atherosclerotic plaques. Circulation 123: 989-998. doi:10.1161/CIRCULATIONAHA.110.984146. PubMed: 21339485. 21339485 10.1161/CIRCULATIONAHA.110.984146 PMC3131163

[46] Cardilo-Reis L, Gruber S, Schreier SM, Drechsler M, Papac-Milicevic N et al. (2012) Interleukin-13 protects from atherosclerosis and modulates plaque composition by skewing the macrophage phenotype. EMBO Mol Med 4: 1072-1086. doi:10.1002/emmm.201201374. PubMed: 23027612. 23027612 10.1002/emmm.201201374 PMC3491837

